# Assessment of Candidate Reference Genes for Gene Expression Studies Using RT-qPCR in *Colletotrichum fructicola* from Litchi

**DOI:** 10.3390/genes14122216

**Published:** 2023-12-14

**Authors:** Dingming Dong, Rong Huang, Yuzhuan Hu, Xinyan Yang, Dagao Xu, Zide Jiang

**Affiliations:** 1 Guangdong Province Key Laboratory of Microbial Signals and Disease Control, South China Agricultural University, Guangzhou 510642, China; gddongdingming@163.com (D.D.); 13797509760@163.com (R.H.); 2 Department of Plant Pathology, South China Agricultural University, Guangzhou 510642, China; 13640111730@163.com (Y.H.); yxinyan333@163.com (X.Y.)

**Keywords:** litchi, *Colletotrichum fructicola*, reference gene, gene expression, RT-qPCR

## Abstract

Litchi (*Litchi chinensis* Sonn.) is a tropical fruit originating from southern China that is currently cultivated in subtropical and tropical regions worldwide. Litchi anthracnose, caused by *Colletotrichum fructicola*, a dominant species of *Colletotrichum* spp., is an important disease of litchi that damages the fruits in fields and in post-harvest storage. Real-time quantitative PCR (RT-qPCR) is a common technique with which to detect the expression of and function of target genes quickly and precisely, and stable reference genes are crucial. However, there is no comprehensive information on suitable reference genes of *C. fructicola* present. Here, we designed eight candidate genes (*GAPDH*, *α-tubulin*, *18S*, *β-tubulin*, *EF1a*, *TATA*, *RPS5*, and *EF3*) using *RefFinder* software (programs: *geNorm*, Δ*Ct*, *BestKeeper*, and *NormFinder*) to investigate their reliability in the detection of *C. fructicola* under five different treatments (fungal development stage, temperature, UV, culture medium, and fungicide). The results showed the optimal reference genes under different conditions: *EF1a* and *α-tubulin* for developmental stage; *α-tubulin* and *β-tubulin* for temperature; *α-tubulin* and *RPS5* for UV treatment; *RPS5* and *α-tubulin* for culture medium; *α-tubulin*, *GAPDH*, and *TATA* for fungicide treatments. The corresponding expression patterns of *HSP70* (Heat shock protein 70) were significantly different when the most and the least stable reference genes were selected when treated under different conditions. Our study provides the first detailed list of optimal reference genes for the analysis of gene expression in *C. fructicola* via RT-qPCR, which should be useful for future functional studies of target genes in *C. fructicola*.

## 1. Introduction

Litchi (*Litchi chinensis* Sonn.), a native fruit originating from southern China, has been extensively cultivated in various subtropical and tropical regions for its delicious taste and medicinal value [1]. Litchi anthracnose is an important disease, which can cause flower and fruit drop, as well as fruit rot, thereby severely impacting the litchi industry. *Colletotrichum fructicola* is the dominant pathogenic fungus in *Colletotrichum* spp. causing litchi anthracnose, accounting for more than 70% of cases (our unpublished data). *Colletotrichum* is a widely distributed fungal genus that can cause a variety of plant diseases [2]. Recognized as one of the top 10 plant pathogenic fungi worldwide in the “2016 Global Plant Status Assessment Report”, *Colletotrichum* significantly affects plants globally [3]. Statistics show that fruits exported from the tropical, subtropical, and Mediterranean regions are seriously affected by *Colletotrichum* spp., resulting in economic losses exceeding 80% without effective control measures [4]. Given the impact of *Colletotrichum* spp. on the economic benefits of fruits, researchers have conducted extensive research on various aspects of its biology, including fungal–plant interaction, genomics and genetics, pathogen infection, cell biology, fungicide resistance, and virulence factors [2,5,6]. At the same time, *C. fructicola* has also been identified as the dominant species causing bitter rot in apple [7], mango anthracnose [8], blueberry anthracnose [9], and strawberry anthracnose [10]. Chemical treatment is the primary method for the field control of anthracnose. Benzimidazole fungicides thiophanate-methyl, methoxy-acrylates fungicides azoxystrobin and pyraclostrobin, demethylation inhibitor prochloraz, protective fungicide chlorothalonil, and multi-site fungicide mancozeb are considered effective fungicides for anthracnose control [11]. However, the frequent use of fungicides has resulted in the development of severe fungicide resistance in *C. fructicola*, to the extent that this fungus is deemed to be a pathogen with a moderate risk of resistance to fungicides (http://www.frac.info (accessed on 10 December 2023), Pathogen Risk List). Previous studies have indicated that the resistance of pathogenic fungi to fungicides is primarily caused by single-nucleotide mutations in target genes, which led to a change in amino acids [12]. Furthermore, changes in the expression of related genes can play a crucial role in the development of fungicide resistance in pathogenic fungi [13,14,15,16]. However, there are no “universal” reference genes that are stably expressed under all possible test conditions [17]. Therefore, the selection of suitable and reliable reference gene is fundamental to the expression research of genes involving fungicide resistance in *C. fructicola*. In addition, lab experiments are indispensable for better understanding the growth and development, pathogenicity mechanism, and functional genomics research of *C. fructicola*, which makes the assessment of the stability of reference genes’ exposure to different lab treatments in *C. fructicola* necessary.

Understanding transcriptional regulation and gene function characterization is based on the analysis of transcript levels of specific genes [18]. RT-qPCR (real-time quantitative polymerase chain reaction) serves as a powerful tool with which to quantify gene expression and offers multiple advantages such as convenience, high sensitivity and specificity, wide application, and so on [19]. Gene expression profiles can also be assessed through the application of RT-qPCR [20]. RT-qPCR has been currently used for gene expression analysis in animals, bacteria, viruses, and fungi [20]. The reference genes in RT-qPCR research are needed for the normalization of data to eliminate or minimize technical variances among the tested samples and accurately estimate the expression of the target genes [21,22]. To identify the most suitable reference genes, it is necessary to systematically design experiments to assess the stability of various candidate reference genes across different species and experimental conditions. There were five reports of suitable reference genes for RT-qPCR analysis in *Colletotrichum* spp., but only one for those in *C. fructicola* [23,24,25,26,27], which has restricted further investigations into the functional genes of *C. fructicola*. Thus, it is necessary to identify suitable reference genes in *C. fructicola* under different experimental conditions.

A stable reference gene is essential in multiple experimental conditions [28]. Traditionally, reference genes are commonly chosen from housekeeping genes [29]. However, no gene maintains constant and stable expression under all experimental conditions [17]. Hence, it is mandatory to choose appropriate reference genes to accurately assess gene expression patterns under specified condition. Moreover, autophagy, as a highly conserved mechanism, exists in all stages of filamentous fungi, and can enable fungi to conquer diverse stress conditions and facilitate the recycling of cytoplasmic components and organelles. Heat shock protein 70 (HSP70), a heat-responsive stress protein, can prevent cell death and apoptosis after exposure to heat, and plays a crucial role in self-recognition, mycoparasitism, and thermal stress [30,31].

The purpose of this research was to identify the most suitable reference genes for *C. fructicola* in different development periods, temperatures, different durations of UV treatment, culture media, and fungicides. The findings could serve as the basis for future research on the expression of key genes of *C. fructicola* in plant–pathogen interactions and the response to other stresses.

## 2. Materials and Methods

### 2.1. Inoculation of C. fructicola

The isolate GDZJ, isolated from litchi fruits with typical symptoms of anthracnose, was identified molecularly as *C. fructicola*. Litchi fruit was obtained from Zhanjiang City, Guangdong Province, China in 2019. The isolate was incubated on potato dextrose agar (PDA), consisting of 200 g of potato, 20 g of dextrose, 20 g of agar, and 1 L of distilled water, sloped at 4 °C before this experiment.

### 2.2. The Preparation and Collection of Samples

The candidate reference genes of *C. fructicola* were assessed with respect to the stage of development, temperature, culture medium, presence of different fungicides, and UV treatment. Each *C. fructicola* developmental stage was sampled; mycelial plugs obtained from the edge of fresh colonies which were inoculated for 5 days were placed into PDB and onto PDA plates, and then ungerminated conidia were harvested after incubation in PDB at 28 °C for 5 days in darkness, shaking at 150 rpm; mycelia were harvested after incubation for 3 and 7 days on PDA under a photoperiod of 12 h. For the temperature treatment, mycelia plugs were incubated on PDA plates with a temperature of 25 °C for 5 days, followed by 15, 25, and 35 °C for 2 days. For different culture media treatments, mycelial plugs taken from the edge of fresh colonies were placed onto the following media: PDA, AEA (with 20 mL of glycerol, 5 g of yeast extract, 0.25 g of MgSO_4_·7H_2_O, 6 g of NaNO_3_, 1 g of KCl, 1.5 g of KH_2_PO_4_, 20 g of agar, and 1 L of distilled water), Czapek’s medium (6 g of NaNO_3_, 1 g of K_2_HPO_4_, 0.5 g of MgSO_4_·7H_2_O, 0.5 g of KCl, 0.01 g of FeSO_4_, 30 g of sucrose, 20 g of agar, and 1 L of distilled water), and YBA (10 g of peptone, 10 g of yeast extract, 0.5 g of sodium acetate, 20 g of agar, and1 L of distilled water). After 5 days of incubation in a biological incubator at 28 °C with a photoperiod of 12 h, mycelia of each treatment were collected. For different fungicide treatments, the effective concentrations for the 50% inhibition (EC_50_) of prochloraz, azoxystrobin, pyraclostrobin, thiophanate-methyl, chlorothalonil, and mancozeb in *C. fructicola* were estimated based on mycelial linear growth rates. Then, mycelia were collected after being treated with different fungicides for 5 days by transferring 5 mm agar plugs of actively growing mycelia to the center of PDA plates, to which were added prochloraz, azoxystrobin, pyraclostrobin, thiophanate-methyl, chlorothalonil, and mancozeb at final concentrations of the respective EC_50_ values. For UV irradiation treatments, 5-day-old mycelia cultured on PDA plates were exposed to UV radiation at distance of 30 cm from a stable UV lamp (30 W; 254 nm) for 30, 90, and 150 s. After irradiation, the mycelia were immediately placed in a dark environment for 1 h and then collected. All treatments were repeated three times.

### 2.3. The Extraction of Total RNA and the Synthesis of cDNA

All-In-One DNA/RNA Mini-Preps Kit (Sangon, Shanghai, China) was used to extract total RNA from different samples of *C. fructicola*. NanoDrop One spectrophotometer (Thermo Fisher Scientific, Waltham, MA, USA) was used to determine RNA concentration and purity. RNA integrity was evaluated via 1% agarose gel electrophoresis. As directed by the manufacturer, 1 g of RNA from each sample was used to synthesis cDNA using PrimeScript^TM^ RT Reagent Kit (Takara, Kyoto, Japan). The concentration of cDNA was determined and diluted to 100 ng/µL before the RT-qPCR.

### 2.4. The Design of Primers and the Cloning of Genes

Eight candidate genes (*GAPDH*, *α-tubulin*, *18S*, *β-tubulin*, *EF1a*, *TATA*, *RPS5*, and *EF3*) have been reported as suitable reference genes for qPCR and were used in this study [32,33,34,35]. According to recently sequenced transcriptomes for *C. fructicola*, specific primers (Table 1) were designed with Primer Premier 5 Tool. All primers met the following parameters: the amplicon length was between 75 and 250 bases; the length of the primers was 20 ± 2 bases; the annealing temperature (Tm) was between 55 °C and 60 °C, and the GC content was at about 50%. PCR was performed with a 25 µL reaction mixture containing LA Taq DNA polymerase (Takara, Japan). In accordance with previous studies, PCR settings were employed [36]. Each PCR product was purified and inserted into the vector pMD18-T (Takara, Japan) for sequencing. To confirm all reference genes’ sequences, the National Center of Biotechnology Information (NCBI) database was used.

### 2.5. RT-qPCR

The RT-qPCR reaction was performed utilizing SYBR Green PCR Mix (2×) Kit (Takara, Japan) in the CFX96 real-time PCR system (Bio-Rad, Hercules, CA, USA). In a 50 µL reaction mix, 2.5 µL of each primer was mixed with 25 µL of SYBR Green Premix (Takara, Japan), 2.5 µL of diluted cDNA (about 100 ng/µL), and 17.5 µL of RNase-free water. In the following step, three technical replicates were prepared, each containing approximately 15 µL of solution. The RT-qPCR program started with a denaturation step at 95 °C for 3 min, and after being subjected to a temperature of 95 °C for 10 s and 55 ° C for 30 s, the process continued for 40 cycles. A comprehensive analysis of the melting curve took place following the completion of the PCR run, spanning temperatures from 55 to 95 °C, increasing the temperature gradually by 0.5 °C every 10 s. Three technical replicates of the samples were employed in this study. Based on linear regression models, slope analysis was used to calculate the RT-qPCR for each gene. The diluted conidia cDNA samples (5^−1^, 5^−2^, 5^−3^, 5^−4^, and 5^−5^) were utilized to construct standard curves for the purpose of determining gene amplification efficiency (*E*).

$$E=\left(10^{\left(-\frac{1}{slope}\right)}-1\right)\times 100.$$

### 2.6. Assessment of Gene Expression Stability

By using the website RefFinder (http://blooge.cn/RefFinder/ (accessed on 23 August 2023)), which offers four automatic programs for analysis, *geNorm* [37], *NormFinder* [38], *BestKeeper* [39], and the ∆*Ct* method [40], each reference gene’s stability was assessed. In the *geNorm* program, pairwise variation (Vn/Vn+1) was computed for the standardization factors NFn and NFn+1. If the calculated Vn/Vn+1 value was equal to or less than 0.15, it indicated that the optimal number of reference genes for the RT-qPCR analysis was n.

Absolute quantification was used to determine the copy number of *HSP70* under different temperature treatments. The RT-qPCR primer containing the *HSP70* gene fragment was designed, (forward primer: 5′-CCCCTCTTTCCCTCGGTAT-3′; reverse primer: 5′-GCTGGTTGTCGGAGAAGGTA-3′). The PCR product was purified and inserted into the vector pMD18-T (Takara, Japan) and then the recombinant plasmid DNA was extracted. The plasmid copy numbers were calculated using the following formula: Plasmid copy number (copy number/μL) = [6.02 × 1023 × plasmid concentration (ng/μL) × 10 − 9]/[plasmid length × 660]. Plasmid DNA was diluted in a 5-fold gradient for the preparation of calibration curves. For better accuracy, each sample in the dilution series was run in triplicate.

To test the stability of selected reference genes, *HSP70* from *C. fructicola* was used as the target gene. Target gene expression was validated and quantified under different treatment conditions. Gene expression was determined by using the forward primer (5′–3′) ACCTTCTCCACCGTCATCTG and reverse primer (5′–3′) ATTGTGAGATTGCCGACGA. The quantification of *HSP70* expression in various treatments was determined by normalizing it to that of the two or three genes with the highest stability and the lowest stability.

### 2.7. Statistical Analysis

Using the 2^−^^∆∆*Ct*^ method, *HSP70*’s relative expression pattern was calculated [41]. SPSS software (v18.0) (SPSS Inc., Chicago, IL, USA) was used to conduct a one-way analysis of variance for all treatments in order to detect significant differences in *HSP70* expression levels.

## 3. Results

### 3.1. Detection of Primer Specificity and Efficiency

**Figure 1:**
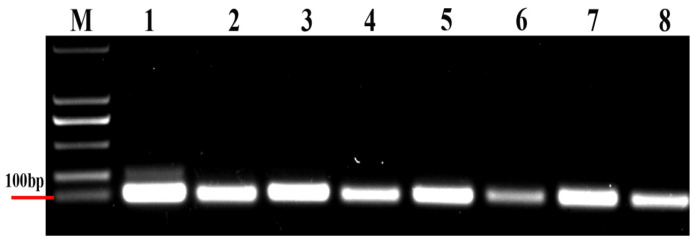
Electrophoresis of non-specific PCR products of the eight candidate reference genes on agarose gel. M, DL2000 DNA marker. PCR products are as follows: (1) *GAPDH*; (2) *α-tubulin*; (3) *18S*; (4) *β-tubulin*; (5) *EF1a*; (6) *TATA*; (7) *RPS5*; and (8) *EF3*.

According to the agarose gel electrophoresis of the PCR products, all the primers amplified a single fragment of the eight selected reference genes from the DNA template (Figure 1); the size of each DNA fragment is shown in Table 1.

**Figure 2:**
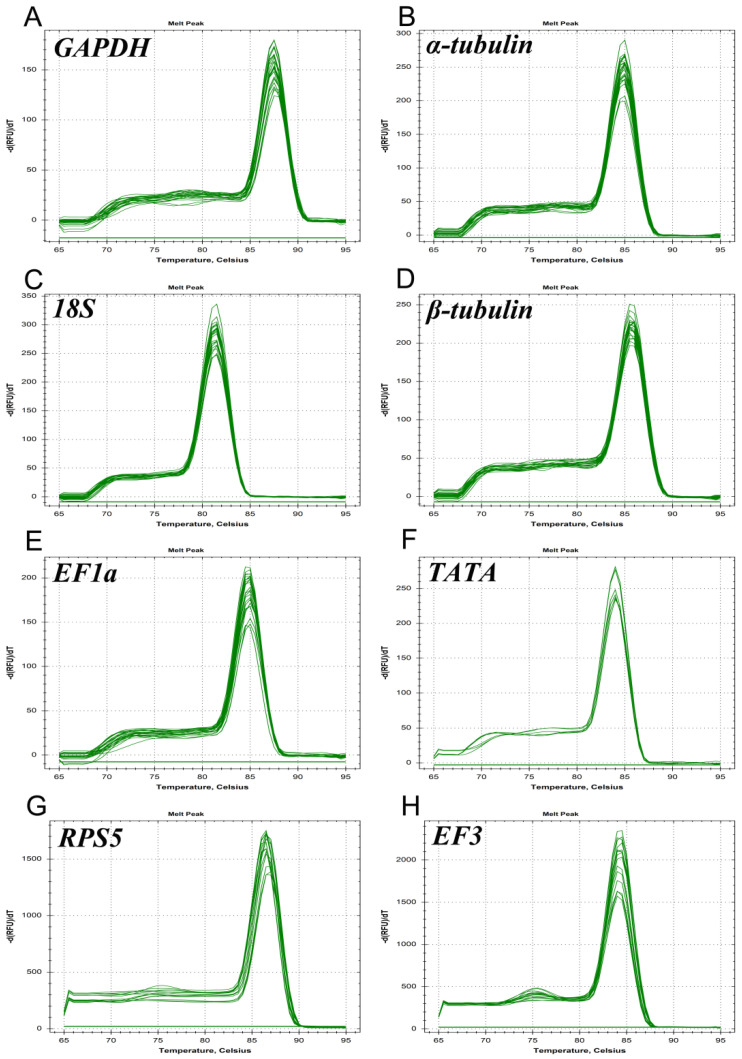
Melting curve analysis of 8 candidate reference genes. Melting curve analysis of (**A**) *GAPDH*; (**B**) *α-tubulin*; (**C**) *18S*; (**D**) *β-tubulin*; (**E**) *EF1a*; (**F**) *TATA*; (**G**) *RPS5*; and (**H**) *EF3*.

All eight reference genes were expressed in *C. fructicola*, and the melting curve analysis indicated that a single peak was produced by each pair of primers, with no non-specific bands (Figure 2).

**Figure 3:**
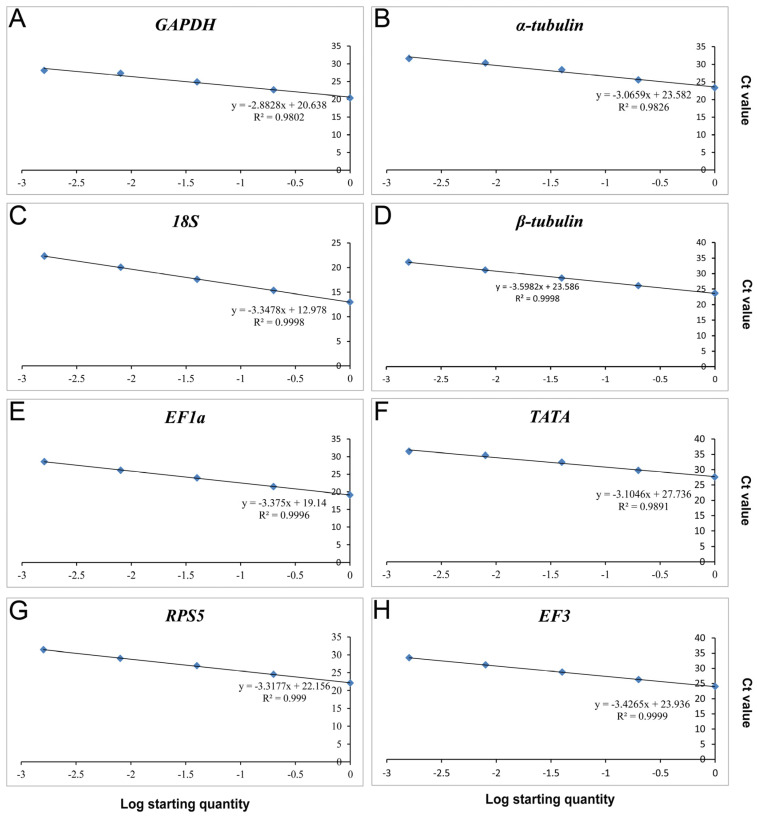
Standard curves of the eight candidate genes. Standard curves of of (**A**) *GAPDH*; (**B**) *α-tubulin*; (**C**) *18S*; (**D**) *β-tubulin*; (**E**) *EF1a*; (**F**) *TATA*; (**G**) *RPS5*; and (**H**) *EF3*.

We constructed a standard curve (Figure 3) encompassing all R^2^ of primers that exhibited a correlation coefficient exceeding 0.98, while maintaining an amplification efficiency (*E*) value ranging from 89.63% to 122.27% (Table 1). The equations for linear regression are presented in Table 1.

### 3.2. Expression Levels of Candidate Reference Genes

**Figure 4:**
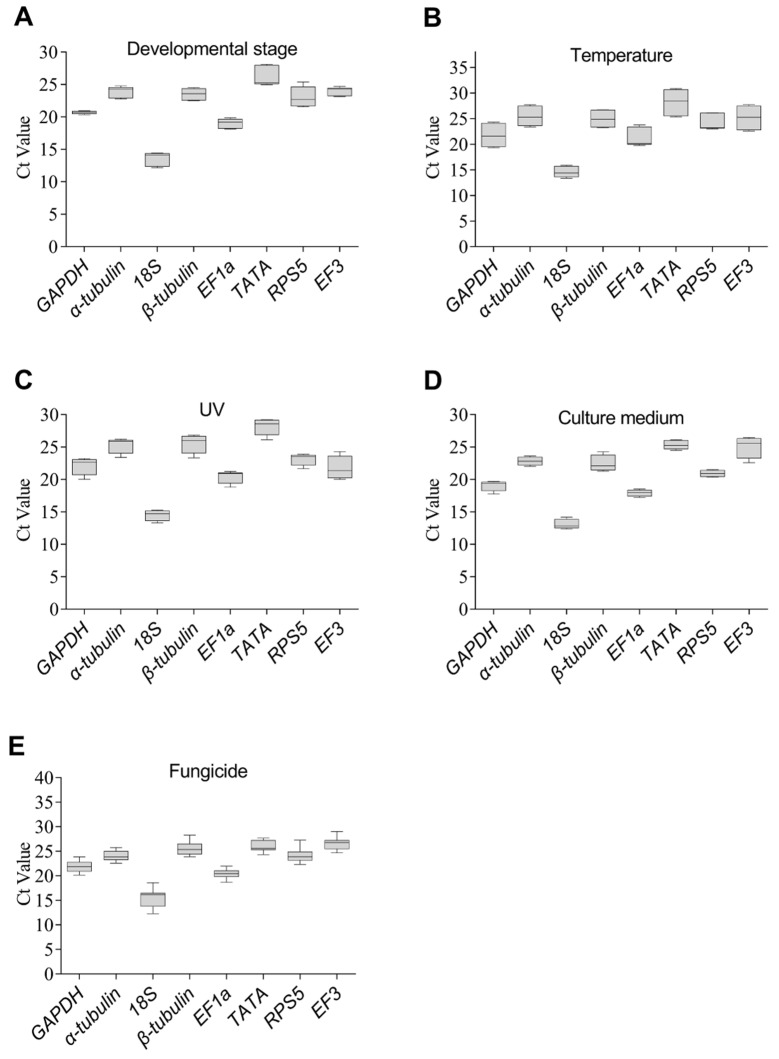
Box and whisker plots of Ct values for eight candidate reference gene across five experimental conditions. (**A**) different developmental stages, (**B**) different temperatures, (**C**) different times treated by UV, (**D**) different culture media, and (**E**) different fungicides. All the independent experiments were repeated three times. Boxes represent lower and upper quartiles of cycle thresholds range with medians indicated, and whisker caps represent maximum and minimum values. About 75 ng of cDNA was added for each experiment.

The analysis of the Ct values of the eight selected candidate genes under five experimental conditions showed that Ct values for eight candidate genes ranged from 12.2 to 30.95 (Figure 4). In addition, *18S* exhibited the lowest Ct values, indicating that it was the gene with the highest expression across all experimental scenarios. Except for individual genes under specific circumstances, Ct values for the remaining seven candidate reference genes were mostly around 23.

### 3.3. The Stability of the Eight Reference Genes under Specific Experimental Conditions

Under the different experimental conditions, the Δ*Ct*, *bestkeeping*, *geNorm*, and *NormFinder* tools were used to assess the stability of the eight candidate reference genes (Table 2).

**Figure 5:**
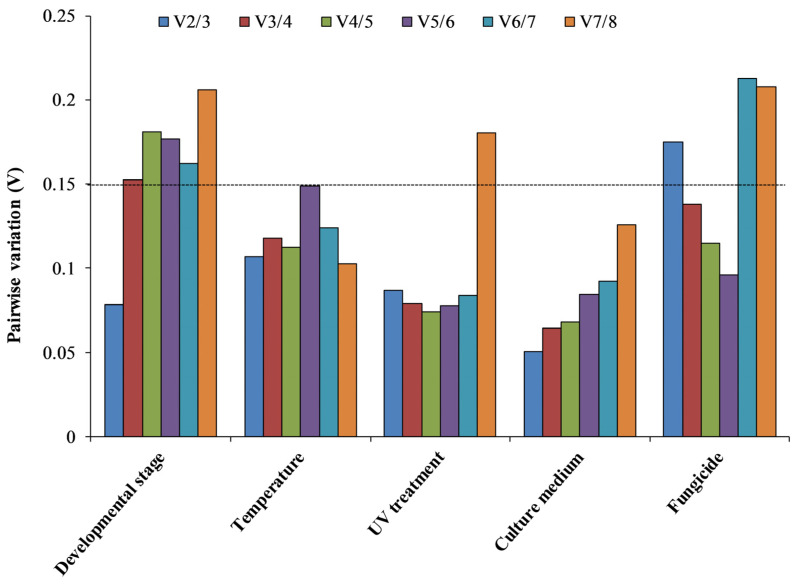
Optimal reference gene numbers for effective normalization. Pairwise variation (V) in the candidate reference genes calculated via *geNorm* based on different comparisons: developmental stage, temperature, UV, culture medium, and fungicide.

*geNorm*. To assess the minimum quantity number of candidate genes for optimal normalization, the pairwise variation was computed with *geNorm* (Figure 5). The results showed that initial V-value (V2/3) was less than 0.15 in four experimental conditions (developmental stage, temperature, UV and culture medium), which indicated that the quantity of two reference genes was adequate for optimal normalization under various experimental conditions. For fungicide experimental settings, the initial V-value (V3/4) was less than 0.15. *β-tubulin* and *EF1a*; *GAPDH* and *EF3*; *α-tubulin* and *EF1a*; *EF1a* and *RPS5*; and *α-tubulin*, *β-tubulin,* and *TATA* were considered to be optimal for the development stage, UV treatment, temperature, culture medium, and fungicide treatment, respectively (Table 2).

*NormFinder*. *NormFinder* analysis identified the most stable reference gene for each condition: *EF1a* for fungal development stage, *α-tubulin* for temperature and culture medium, *RPS5* for UV treatments, and *GAPDH* for fungicide comparisons.

*BestKeeper*. In the fungal developmental stages, the standard deviation values for *RPS5* and *TATA* exceeded 1, excluding them from being utilized as reference genes. Except for *18S*, none of the other genes could be used to research temperature variations. The genes *GAPDH*, *β-tubulin*, and *EF3* should not be used to research UV treatment variations. Moreover, it is not advisable to consider *β-tubulin* and *EF3* reference genes when comparing culture mediums. Additionally, *GAPDH*, *EF3*, *RPS5*, *β-tubulin*, and *18S* are unsuitable as reference genes for fungicide differences. The best gene for developmental stage was *GAPDH*, and that for culture medium comparisons and fungicide was *EF1a*. However, in comparisons of temperature and UV treatment, *18S* was determined to be the most stable gene.

Δ*Ct* method. When considering fungal development stages, *EF1a* was the best reference gene, while *α-tubulin* was the best gene for temperature, UV treatment, and culture medium-related experiments. For fungicide treatment, *TATA* was most stable reference gene.

### 3.4. The RefFinder Ranking for Eight Reference Gene Expression Stability

**Figure 6:**
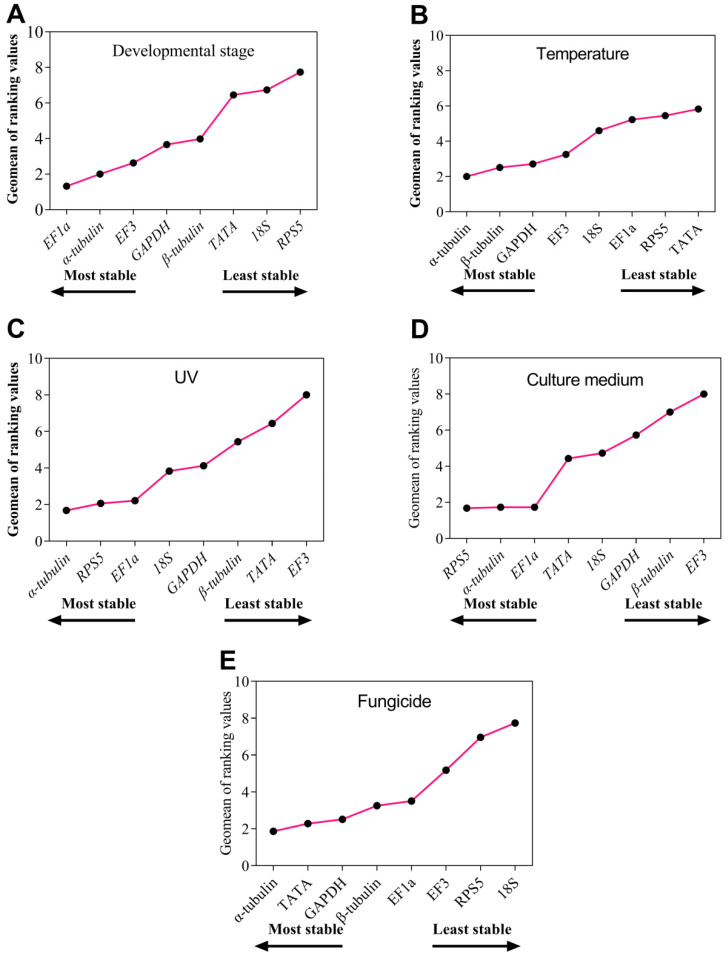
Stability of the expression of the eight candidate reference genes in *C. fructicola* under different conditions analyzed with *RefFinder*. Stability of the expression of the eight candidate reference genes under different (**A**) developmental stage, (**B**) temperatures, (**C**) durations of UV treatment, (**D**) culture media, and (**E**) fungicides. A lower Geomean value means a more stable expression.

After the analysis of the available reference genes, *RefFinder* comprehensively ranked the reference genes for fungal development stage as *EF1a* > *α-tubulin* > *EF3* > *GAPDH* > *β-tubulin* > *TATA* > *18S* > *RPS5* (Figure 6A). Similarly, the ranking for temperature was *α-tubulin* > *β-tubulin* > *GAPDH* > *EF3* > *18S* > *EF1a* > *RPS5* > *TATA* (Figure 6B), whereas that for UV treatment was *α-tubulin* > *RPS5* > *EF1a* > *18S* > *GAPDH* > *β-tubulin* > *TATA* > *EF3* (Figure 6C). For culture medium samples, the ranking was *RPS5* > *α-tubulin* > *EF1a > TATA > 18S > GAPDH > β-tubulin > EF3* (Figure 6D), whereas it was *α-tubulin* > *TATA* > *GAPDH* > *β-tubulin > EF1a > EF3 > RPS5 > 18S* under different fungicide conditions (Figure 6E).

### 3.5. Evaluation of the Selected Reference Genes

**Figure 7:**
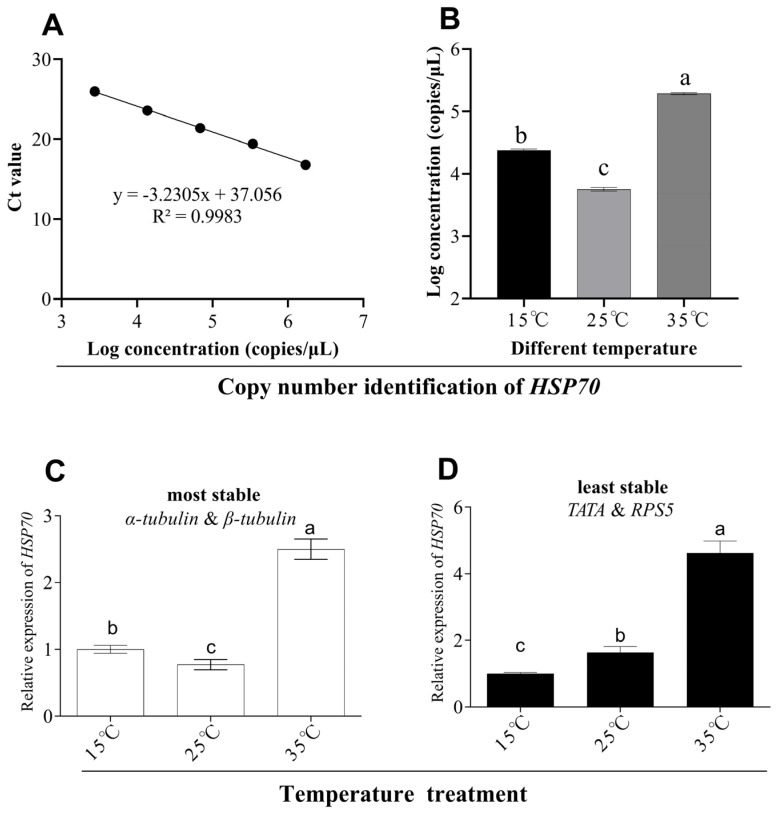
The expression of *HSP70* under temperature treatment. Standard curves of *HSP70* gene (**A**). The copy number of *HSP70* under different temperature treatments (**B**). The relative abundance of *HSP70* under different temperatures normalized to that of the best genes (*α-tubulin* and *β-tubulin*) (**C**) and worst genes (*RPS5* and *TATA*) (**D**). The values are means ± standard error. Significant differences are indicated by different lowercase letters (*p* < 0.05).

In order to determine the copy number of *HSP70* under different temperature treatments, a standard curve of the *HSP70* gene was made (Figure 7A) and the copy number of *HSP70* under different temperature treatments was determined from the standard curve. Based on the results, we learnt that *HSP70* has a higher copy number at both low and high temperatures as compared to that under 25 °C conditions (Figure 7B). Similarly, when we calculated the relative expression of *HSP70* using the most stable reference gene, this was consistent with the above results (Figure 7C). However, when the least stable reference gene was utilized, inconsistent results were obtained (Figure 7D).

**Figure 8:**
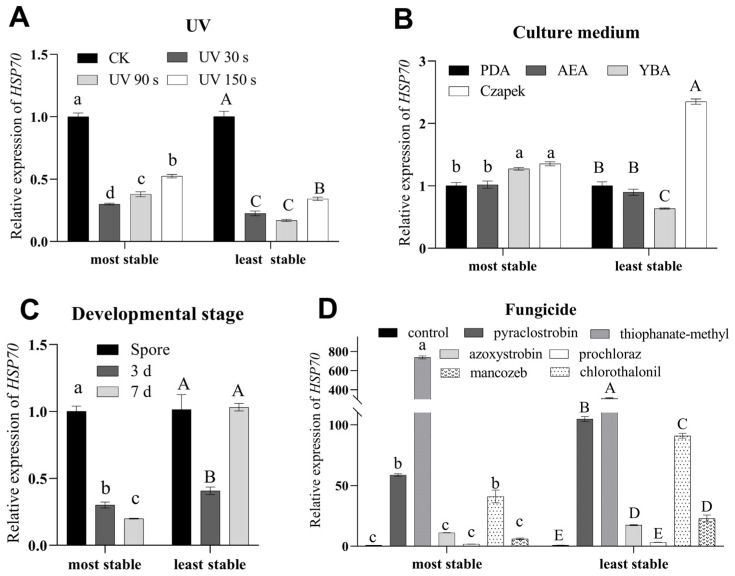
The relative expression of *HSP70* in different treatments. The relative expression of *HSP70* under UV treatment times (**A**) was normalized to that of the most stable (*α-tubulin* and *RPS5*) and least stable (*TATA* and *EF3*) reference genes. CK means that the UV treatment time was 0. The relative expression of *HSP70* for culture media (**B**) was normalized to that of the best genes (*RPS5* and *α-tubulin*) and worst genes (*β-tubulin* and *EF3*), respectively. The relative expression of *HSP70* in developmental stages (**C**) was normalized to that of the best genes (*EF1a* and *α-tubulin*) and worst genes (*18S* and *RPS5*), respectively. The relative expression of *HSP70* treated using different fungicides (**D**) was normalized to that of the best genes (*α-tubulin*, *TATA* and *GAPDH*) and worst genes (*EF3*, *RPS5*, and *18S*). Control means that fungi were cultured without fungicides. The values are means ± standard error. Significant differences normalized to the most stable are indicated by different lowercase letters (*p* < 0.05). Significant differences normalized to the least stable are indicated by different uppercase letters (*p* < 0.05).

To assess and contrast the stability of chosen reference genes, we focused on the expression among all the different treatments with *HSP70*. However, under different development stages, UV treatments, culture media, and fungicides, the expression pattern and relative expression of the *HSP70* gene was different between the most stable and the least stable reference genes (Figure 8A–D).

## 4. Discussion

Our study focused on the identification of the most suitable reference genes for *C. fructicola* in different development periods, temperatures, different durations of UV treatment, culture media, and fungicides via the evaluation of the stability of eight candidate genes, including *GAPDH*, *α-tubulin*, *18S*, *β-tubulin*, *EF1a*, *TATA*, *RPS5*, and *EF3*. The stability of the eight genes was evaluated in five experimental conditions using *RefFinder* software including *geNorm*, ∆*Ct*, *BestKeeper* and *NormFinder* programs. Considering its important role in autophagy, a conserved mechanism in all stages of fungus development, *HSP70*, was used as a reporter gene. The stability of potential reference genes of *C. fructicola* causing litchi anthracnose has been systematically evaluated for the very first time.

RT-qPCR has emerged as a pivotal method through which to analyze gene transcript profiles owing to its sensitivity, accuracy, and reproducibility [19,42,43]. To accurately determine the expression pattern of a target gene using RT-qPCR, an appropriate reference gene must be selected. The expression of reference genes is expected to remain stable and consistent regardless of time or different experimental conditions [19,42,43]. Without a primary investigation of their stability, using previously identified reference genes for the normalization of target gene expression under specific experimental conditions could result in significant issues and inaccuracies in the data and their interpretation [44]. Hence, it is crucial to validate the chosen reference genes under specific experimental conditions in order to address this concern.

As a dominant disease on litchi, litchi anthracnose severely impacted the litchi industry. A comprehensive understanding of the pathogenesis and fungicide resistance mechanisms of pathogenic fungi that cause such disease requires the study and elucidation of the role of genes and variations in gene expression under different conditions [45,46,47]. In the past few years, reports on the screening of reference genes in plant pathogenic fungi have been on the rise [17,40,48], but the reference genes of the litchi anthracnose pathogen have been largely neglected to date. Therefore, eight reference genes (*GAPDH*, *α-tubulin*, *18S*, *β-tubulin*, *EF1a*, *TATA*, *RPS5*, and *EF3*) were selected and evaluated in different experimental treatments in this study.

Reference genes need consistent high-level expression under diverse conditions. Briefly, *18S* is generally considered to have high expression in various experimental conditions [36,44], and the remaining seven candidate reference genes are also highly expressed under various conditions and have been commonly used as reference genes [28,36,43,44]. Therefore, these eight genes have the potential to serve as reference genes in *C. fructicola* studies. After evaluating the Ct value in RT-qPCR, we initially examined the expression of the candidate reference genes in this research. The Ct values of the eight potential reference genes were approximately 23 for the majority of the tested samples. Additionally, the linear amplification of all candidate reference genes was exceptional, as demonstrated by R2 values surpassing 98%. The amplification efficiency met the basic requirements of 89.63–122.27% [28].

The appropriateness of reference genes for transcript normalization was determined using Δ*Ct*, *geNorm*, *NormFinder*, and *BestKeeper* programs during fungal development stage, temperature, UV, culture medium, and fungicide conditions in *C. fructicola* [48,49]. Taken together, the rankings of the optimal reference genes derived from various conditions and software analyses were similar to the results of previous studies [44]. Previous studies have highlighted the importance of selecting multiple reference genes for unbiased normalization in RT-qPCR analysis [28]. The required number of reference genes for proper normalization may differ based on the specific experimental conditions [44]. Figueiredo determined that three reference genes were sufficient for the entire dataset, while four reference genes were sufficient for Catimor 88 and two or three reference genes were sufficient for Caturra hypocotyls inoculated with *Colletotrichum kahawae* [43]. However, utilizing an excessive number of reference genes may create practical challenges during the execution and operation of a study [24]. Therefore, to ensure reliable normalization, it is optimal to choose a minimum of two highly accurate reference genes, and this has been confirmed in previous studies [48,50]. In this study, we utilized geNorm program to perform pairwise analysis, confirming that for assessing gene expression levels via RT-qPCR during fungal development or under different temperature, UV, or culture medium conditions, at least two out of the eight genes could serve as validated reference genes. On the other hand, three reference genes were adequate for normalization under fungicide conditions. Based on *RefFinder*, *EF1a* and *α-tubulin* are the most stable genes of *C. fructicola* for developmental stage, *α-tubulin* and *β-tubulin* are the most stable for for different temperatures, *α-tubulin* and *RPS5* are the most stable for different UV treatments, *RPS5* and *α-tubulin* are the most stable for different culture media, and *α-tubulin*, *TATA*, and *GAPDH* are the most stable for different fungicides.

*Tubulin* (*β-tubulin* and *α-tubulin*) served as a reference gene in numerous research studies [44]. For example, *β-tubulin* was used as a reference gene in the manipulation of temperature conditions [48,50], which produced results consistent with ours. Furthermore, the level of expression for *a-tubulin* remains consistent across various tissues and sexes of *Coleomegilla maculata* [51], and *a-tubulin* was identified as the most stable reference gene for diverse UV exposure, temperature, and fungicide treatment experiments in this study. It is widely acknowledged that ribosomal protein (RP), a highly conserved protein in all living organisms, plays a crucial role as a fundamental constituent of ribosomes. Previous research has consistently identified RP-encoding genes (like *RPL* and *RPS*) as optimal reference genes for studying gene expression. For example, in *C. kahawae* samples, *RPL18* was revealed served as the optimal reference gene [24]. Based on our results, the stability of *RPS5* as a reference gene in experiments related to different culture mediums is unparalleled. However, it is the least stable reference gene for fungal development stage experiments. *EF1a*, involved in protein synthesis, is widely used for normalizing gene expression. For example, *EF1a* showed the highest level of stability among the reference genes in *Aphis craccivora* regardless of the developmental stages or temperature treatments [44]. Our findings indicate that *EF1a* was consistently stable across various development stages. Overall, our results provide additional support for the need to validate the suitability of frequently utilized reference genes under distinct experimental circumstances.

In order to confirm the reliability of the potential reference genes found in our study, we evaluated the expression profile of the target gene *HSP70* using a selection of the most consistent reference genes and the least consistent reference genes among all the different treatments. However, under different development stages, UV, culture media, and fungicides, the expression pattern and relative expression of the *HSP70* gene were different between the use of the most stable and the least stable reference genes. The results illustrated the importance of selecting the appropriate reference gene when performing RT-qPCR analysis, since the utilization of unreliable reference genes led to imprecise or insignificant variations in transcript levels. Thus, in order to ensure accurate analyses of RT-qPCR data in *C. fructicola*, it is essential to utilize a dependable reference gene.

## 5. Conclusions

Eight candidate reference genes of *C. fructicola* were selected and their stability was evaluated under five experimental conditions. To our knowledge, this is the first report of a comparison and identification of RT-qPCR reference genes for *C. fructicola* in five distinct experimental conditions. The findings should facilitate future analyses of target gene expression profiles in *C. fructicola*, which should prove useful for functional studies of the target genes in *C. fructicola* from litchi.

## Figures and Tables

**Table 1:** Information about the candidate reference genes in a nutshell.

Reference Genes	Forward/Reverse Primer Sequence (5′–3′)	Amplicon Size (bp)	Efficiency (%)	R^2^	Linear Regression
*GAPDH*	F: GTCTCCGTCGTTGACCTGAC	120	122.27	0.9802	y = −2.8828x + 20.638
	R: GTCCTCAGTGTAGGCCAGGA				
*α-tubulin*	F: CTCAGGTCGTCTCCTCCATC	110	111.92	0.9826	y = −3.0659x + 23.582
	R: GGGGTAGTGGATACGAGGGT				
*18S*	F: CAGGAACGAAAGTTAGGGGA	120	98.93	0.9998	y = −3.3478x + 12.978
	R: ATTTCTCGTAAGGTGCCGAA				
*β-tubulin*	F: CTGCCTTCTGGCAAAACATT	108	89.63	0.9998	y = −3.5982x + 23.586
	R: GCTTCGTTGAAGTAGACGCTC				
*EF1a*	F: CCAACGTCACCACTGAAGTC	109	97.83	0.9996	y = −3.375x + 19.14
	R: TCCTTGACGGAGACGTTCTT				
*TATA*	F: TTCGCCTCTGGTAAGATGGT	120	109.94	0.9891	y = −3.1046x + 27.736
	R: GAAGTCGGTGAACTTGGCAT				
*RPS5*	F: CAACTGCCCCATCATTGAG	119	100.18	0.9990	y = −3.3177x + 22.156
	R: TCATCAGGTGGATGATCTCG				
*EF3*	F: GGACACCAAGAAGGAGGTCA	109	95.81	0.9990	y = −3.4265x + 23.936
	R: ACACTTGATAAGCTCGGGGA				

**Table 2:** The stability of gene expression assessed using *geNorm*, *Normfinder*, *BestKeeper*, and Δ*Ct* for eight candidate reference genes of *C. fructicola* under different experimental conditions.

Conditions	Rank	*geNorm*		*NormFinder*		*BestKeeper*		Δ*Ct*	
		Gene	Stability	Gene	Stability	Gene	Stability	Gene	Stability
Developmental stage	1	*β-tublin*	0.142	*EF1a*	0.071	*GAPDH*	0.2	*EF1a*	0.79
	2	*EF1a*	0.142	*β-tubulin*	0.137	*EF3*	0.53	*β-tubulin*	0.83
	3	*α-tubulin*	0.21	*GAPDH*	0.32	*EF1a*	0.59	*α-tubulin*	0.88
	4	*GAPDH*	0.415	*α-tubulin*	0.358	*β-tubulin*	0.66	*GAPDH*	0.93
	5	*EF3*	0.625	*EF3*	0.694	*α-tubulin*	0.71	*EF3*	1.05
	6	*TATA*	0.796	*TATA*	1.13	*18S*	0.86	*TATA*	1.34
	7	*18S*	0.917	*18S*	1.252	*RPS5*	1.23	*18S*	1.39
	8	*RPS5*	1.113	*RPS5*	1.625	*TATA*	1.27	*RPS5*	1.7
Temperature	1	*GAPDH*	0.176	*α-tubulin*	0.137	*18S*	0.83	*α-tubulin*	0.58
	2	*EF3*	0.176	*β-tubulin*	0.174	*β-tubulin*	1.19	*β-tubulin*	0.62
	3	*TATA*	0.276	*GAPDH*	0.376	*RPS5*	1.32	*GAPDH*	0.64
	4	*α-tubulin*	0.381	*EF3*	0.541	*α-tubulin*	1.41	*EF3*	0.7
	5	*β-tubulin*	0.461	*EF1a*	0.672	*EF1a*	1.58	*EF1a*	0.84
	6	*EF1a*	0.617	*RPS5*	0.753	*GAPDH*	1.64	*TATA*	0.88
	7	*RPS5*	0.701	*18S*	0.797	*EF3*	1.67	*RPS5*	0.88
	8	*18S*	0.758	*TATA*	0.81	*TATA*	1.84	*18S*	0.93
UV treatment	1	*α-tubulin*	0.184	*RPS5*	0.117	*18S*	0.66	*α-tubulin*	0.51
	2	*EF1a*	0.184	*α-tubulin*	0.135	*RPS5*	0.69	*EF1a*	0.53
	3	*RPS5*	0.243	*GAPDH*	0.221	*EF1a*	0.8	*RPS5*	0.53
	4	*GAPDH*	0.29	*EF1a*	0.225	*α-tubulin*	0.92	*GAPDH*	0.56
	5	*β-tubulin*	0.332	*β-tubulin*	0.277	*TATA*	0.93	*β-tubulin*	0.61
	6	*18S*	0.387	*18S*	0.323	*GAPDH*	1.04	*18S*	0.65
	7	*TATA*	0.456	*TATA*	0.746	*β-tubulin*	1.08	*TATA*	0.82
	8	*EF3*	0.708	*EF3*	1.439	*EF3*	1.39	*EF3*	1.46
Culture medium	1	*EF1a*	0.113	*α-tubulin*	0.072	*EF1a*	0.46	*α-tubulin*	0.42
	2	*RPS5*	0.113	*RPS5*	0.264	*RPS5*	0.48	*RPS5*	0.46
	3	*α-tubulin*	0.145	*EF1a*	0.268	*α-tubulin*	0.57	*EF1a*	0.48
	4	*TATA*	0.206	*TATA*	0.289	*18S*	0.59	*TATA*	0.49
	5	*18S*	0.267	*18S*	0.384	*GAPDH*	0.61	*18S*	0.55
	6	*GAPDH*	0.353	*GAPDH*	0.4	*TATA*	0.65	*GAPDH*	0.61
	7	*β-tubulin*	0.448	*β-tubulin*	0.574	*β-tubulin*	1	*β-tubulin*	0.73
	8	*EF3*	0.599	*EF3*	0.999	*EF3*	1.22	*EF3*	1.05
Fungicide	1	*α-tubulin*	0.546	*GAPDH*	0.293	*EF1a*	0.8	*TATA*	0.89
	2	*β-tubulin*	0.546	*α-tubulin*	0.302	*α-tubulin*	0.82	*GAPDH*	0.89
	3	*TATA*	0.577	*TATA*	0.32	*TATA*	0.96	*α-tubulin*	0.9
	4	*EF3*	0.609	*β-tubulin*	0.392	*GAPDH*	1	*β-tubulin*	0.9
	5	*GAPDH*	0.639	*EF1a*	0.404	*EF3*	1.08	*EF1a*	0.92
	6	*EF1a*	0.657	*EF3*	0.436	*RPS5*	1.13	*EF3*	0.94
	7	*RPS5*	0.905	*18S*	1.642	*β-tubulin*	1.14	*RPS5*	1.71
	8	*18S*	1.108	*RPS5*	1.648	*18S*	1.47	*18S*	1.72

## Data Availability

Data are contained within the article.
